# Quantifying Emergency Medicine Residency Learning Curves Using Natural Language Processing: Retrospective Cohort Study

**DOI:** 10.2196/82326

**Published:** 2025-12-09

**Authors:** Carl Preiksaitis, Joshua Hughes, Rana Kabeer, William Dixon, Christian Rose

**Affiliations:** 1 Department of Emergency Medicine, Stanford University School of Medicine, 900 Welch Road, Suite 350, Palo Alto, CA, 94304, United States, 1 650-723-6576, 1 650-723-0121

**Keywords:** emergency medicine residency, clinical exposure, learning curves, natural language processing, electronic health records, graduate medical education

## Abstract

**Background:**

The optimal duration of emergency medicine (EM) residency training remains a subject of national debate, with the Accreditation Council for Graduate Medical Education considering standardizing all programs to 4 years. However, empirical data on how residents accumulate clinical exposure over time are limited. Traditional measures, such as case logs and diagnostic codes, often fail to capture the breadth and depth of diagnostic reasoning. Natural language processing (NLP) of clinical documentation offers a novel approach to quantifying clinical experiences more comprehensively.

**Objective:**

This study aimed to (1) quantify how EM residents acquire clinical topic exposure over the course of training, (2) evaluate variation in exposure patterns across residents and classes, and (3) assess changes in workload and case complexity over time to inform the discussion on optimal program length.

**Methods:**

We conducted a retrospective cohort study of EM residents at Stanford Hospital, analyzing 244,255 emergency department encounters from July 1, 2016, to November 30, 2023. The sample included 62 residents across 4 graduating classes (2020‐2023), representing all primary training site encounters where residents served as primary or supervisory providers. Using a retrieval-augmented generation NLP pipeline, we mapped resident clinical documentation to the 895 subcategories of the 2022 Model for Clinical Practice of Emergency Medicine (MCPEM) via intermediate mapping to the Systematized Nomenclature of Medicine, Clinical Terms, Clinical Observations, Recordings, and Encoding problem list subset. We generated cumulative topic exposure curves, quantified the diversity of topic coverage, assessed variability between residents, and analyzed the progression in clinical complexity using Emergency Severity Index (ESI) scores and admission rates.

**Results:**

Residents encountered the largest increase in new topics during postgraduate year 1 (PGY1), averaging 376.7 (42.1%) unique topics among a total of 895 MCPEM subcategories. By PGY4, they averaged 565.9 (63.2%) topics, representing a 9.9% (51/515) increase over PGY3. Exposure plateaus generally occurred at 39 to 41 months, although substantial individual variation was observed, with some residents continuing to acquire new topics until graduation. Annual case volume more than tripled from PGY1 (mean 445.7, SD 112.7 encounters) to PGY4 (mean 1528.4, SD 112.7 encounters). Case complexity increased, as evidenced by a decrease in mean ESI score from 2.94 to 2.79, and a rise in high-acuity (ESI 1‐2) cases from 16% (4374/27,340) to 30.9% (9418/30,466).

**Conclusions:**

NLP analysis of clinical documentation provides a scalable, detailed method for tracking EM residents’ clinical exposure and progression. Many residents continue to gain new experiences into their fourth year, particularly in higher-acuity cases. These findings suggest that a 4-year training model may offer meaningful additional educational value, while also highlighting the importance of individualized assessment given the variability in learning trajectories.

## Introduction

### The Challenge of Measuring Clinical Experience

The optimal duration of emergency medicine (EM) residency training remains a critical unresolved question in graduate medical education. As the Accreditation Council for Graduate Medical Education considers standardizing all programs to a 4-year model, this debate has highlighted a fundamental gap in our understanding. We lack reliable methods to measure how residents accumulate and master clinical experience across a vast spectrum of EM presentations [1]. While these clinical encounters form the foundation of physician development and shape future practice patterns, programs have struggled to systematically track and optimize them, even within competency-based educational frameworks [2-5].

Current methods for measuring clinical exposure in EM suffer from both practical and conceptual limitations. Self-reported case logs, the traditional standard, demonstrate significant error rates due to recall bias [6,7]. Approaches using diagnostic coding systems such as the International Classification of Diseases, 10th revision, fundamentally misalign with EM’s paradigm [8-11]. The core work of emergency physicians, evaluating and ruling out life-threatening conditions, often results in nonspecific final diagnoses (eg, “abdominal pain”) that mask the complexity of care delivered [12]. Furthermore, we know that evaluating for life-threatening illnesses within the context of abdominal pain can be confounded by mimics such as acute coronary syndrome, which are nonspecific, have a high overlap with other conditions, and while they are considered, may not appear in the final diagnosis but nonetheless expose the resident to evaluating for that condition.

### A Novel Natural Language Processing–Based Approach

Natural language processing (NLP) of clinical documentation offers a potential breakthrough. By analyzing the comprehensive narrative content of clinical notes that capture the diagnostic reasoning process, NLP can measure case exposure with greater granularity. When combined with the Systematized Nomenclature of Medicine–Clinical Terms, a clinical terminology system designed to represent complex medical concepts, this approach can systematically document both the breadth of conditions evaluated and the depth of clinical reasoning used. This methodological advance aligns with emerging calls for “precision education” in medical training, where the analysis of clinical data drives personalized learning optimization [5,13].

### Study Objectives

In this study, we used NLP to provide a comprehensive, data-driven analysis of EM resident development. Using electronic health record data from a single academic medical center spanning multiple resident cohorts, we pursued three objectives: (1) quantify topic exposure curves and clinical progression, mapping how residents accumulate diagnostic topic exposure over time; (2) examine variation in clinical exposure patterns between individual residents and graduating classes; and (3) analyze the distribution of clinical experiences across presentation types and complexity levels to provide empirical evidence relevant to the debate on optimal training duration.

## Methods

### Study Design and Population

We conducted a retrospective analysis of emergency department (ED) encounters at Stanford Hospital between July 1, 2016, and November 30, 2023. Stanford Hospital serves as the primary training site for our residency and is a high-volume academic ED and level 1 trauma center. This period captured the primary training site experiences of 4 resident classes (2020‐2023). The study included all EM residents who completed their full 4-year training during this period (n=62). The resident cohort had a mean age of 29.0 (SD 3.6) years, and 40 (64.5%) residents were male. The cohort was predominantly White (n=49, 79%) and non-Hispanic (n=60, 96.8%), with other racial identities including Asian (n=11, 17.7%), Black (n=1, 1.6%), and other (n=1, 1.6%). Encounters in which residents served as either the primary or supervisory resident were included. Resident-patient encounters were identified using the electronic health record’s treatment team data. A rule-based algorithm was developed to attribute each encounter to the appropriate residents. Primary attribution was assigned to the first resident to document their involvement with a patient, and this was cross-referenced with shift schedule data to ensure that the encounter occurred during the resident’s clinical duties. To account for both primary and supervisory roles, encounters were also coattributed to a senior resident if they were documented on the treatment team in close temporal proximity to a junior resident, reflecting an active supervisory role. When both junior and senior residents were assigned to an encounter, we credited topic exposure to both residents for resident-level analysis but counted each encounter once for patient and encounter-level summaries; per–postgraduate year (PGY) proportions used denominators based on unique, non-double-counted encounters. Encounters from nonclinical or administrative shifts were excluded. Our program is a 4-year PGY1 to 4 program where the PGY4 year includes 40 weeks of ED time with a specific emphasis on developing supervisory skills and practicing with graduated responsibility.

Residents also gain clinical experience at 2 high-volume, high-acuity affiliated sites (Kaiser Santa Clara and Santa Clara Valley Medical Center) that use a different electronic health record. These data were not included in our analysis. Depending on the PGY level, these external sites constitute approximately 30% to 35% of a resident’s total ED training time. Encounters from off-service rotations (eg, intensive care unit, anesthesia, and obstetrics) were also excluded. Residents who did not complete the program were excluded from this analysis to ensure the integrity of longitudinal exposure curve construction.

### Ethical Considerations

This study was approved as minimal-risk research by the Stanford University Institutional Review Board (IRB 69107). A waiver of informed consent was granted because the study involved secondary analysis of existing clinical documentation. Data access was authorized through the Stanford Research Repository. All data were deidentified and analyzed within a secure, Health Insurance Portability and Accountability Act–compliant environment, and no identifiable information left the repository. The study complied with institutional and national regulations for human participants research.

### Data Sources and Variables

We extracted deidentified structured data, including patient demographics, Emergency Severity Index (ESI), and disposition status, as well as unstructured data in the form of clinical documentation from the Stanford Research Repository [14]. We focused on note sections capturing resident diagnostic reasoning—history of present illness, medical decision-making, and ED course narratives.

### NLP Overview

We developed a multistage NLP pipeline to map the narrative content of resident clinical documentation to the 895 clinical subcategories of the 2022 Model for Clinical Practice of Emergency Medicine (MCPEM) [15]. Our approach used a retrieval-augmented generation framework with a Health Insurance Portability and Accountability Act–compliant instance of Google’s Gemini 1.5 Flash large language model, which was selected for its balance of cost-effectiveness and high performance within our institution’s available tools [16-18]. This involved extracting key clinical concepts from resident notes and mapping them, as an intermediate step, to the Systematized Nomenclature of Medicine–Clinical Terms, Clinical Observations, Recordings, and Encoding subset before final classification into MCPEM topics [19]. This 2-stage process was chosen to preserve granular clinical detail while using a standardized clinical terminology.

Our retrieval-augmented pipeline can be conceptualized using standard NLP terminology as a 3-stage information retrieval (IR) to information extraction (IE) to classification process. The IR component selects relevant note sections and retrieves candidate concept matches from the Systematized Nomenclature of Medicine–Clinical Terms. The IE component, implemented implicitly through the language model, identifies and normalizes key medical entities and filters them for contextual relevance (eg, negated, historical, or uncertain findings). The classification stage maps these normalized concepts to the MCPEM topics.

### Validation Methodology

We validated this pipeline through a manual review of 500 randomly selected encounters by 4 board-certified emergency physicians (CP, WD, JH, and RK). This process confirmed the high accuracy of our automated approach, with the model’s classifications agreeing with the expert consensus 89.76% (377/420) of the time. The interrater reliability among the physician reviewers was substantial (κ=0.71). A comprehensive description of the NLP architecture, model configuration, and validation methodology is provided in Multimedia Appendix 1.

### Analysis

Our analysis focused on 3 key aspects of resident development. To appropriately account for the resident as the primary unit of analysis and the clustered nature of the data (ie, multiple encounters nested within each resident), all encounter-level data were first aggregated to the individual resident level. Statistical comparisons were performed on this resident-level dataset (N=62).

First, we constructed topic exposure curves by tracking cumulative unique topics over time. Topic exposure rates were calculated using 30-day sliding windows. We defined exposure plateaus as periods where residents encountered fewer than 1 new topic per 100 patients over 3 consecutive measurement windows. Second, we examined variation in exposure by analyzing differences in case volumes, topic coverage, and patient acuity. To quantify the equity of exposure distribution among residents within the same PGY level, we calculated the Gini coefficients. Originally developed to measure income inequality, the Gini coefficient quantifies the inequality of a frequency distribution, with values ranging from 0 (representing perfect equality) to 1 (representing perfect inequality) [20]. In this context, a Gini coefficient of 0 would indicate that all residents in a cohort had the exact same volume of exposure to a given measure (eg, high-acuity cases), while a value of 1 would indicate that a single resident received all of the exposure and all others received none. This metric provides a standardized way to compare the degree of interresident variability across different clinical domains and training years. Third, we tracked clinical complexity progression using ESI scores and admission rates as proxies.

Analyses were performed using R (version 4.3.1; R Foundation for Statistical Computing) and Python (version 3.11). Statistical comparisons between classes were performed using Kruskal-Wallis tests with eta-squared effect sizes. A *P* value of <.05 was considered to indicate statistical significance.

## Results

### Resident Cohort

Our analysis included the primary-site training experiences of 62 EM residents from 4 graduating classes (2020‐2023). Over the course of their training, this cohort managed 244,255 patient encounters, representing 133,748 (54.8%) unique patients. Detailed demographic and clinical characteristics of the patient encounters are provided in Table 1.

**Table 1.** Demographic and clinical characteristics of emergency department patient encounters managed by emergency medicine residents at Stanford Hospital, 2016 to 2023 (N=133,748).

Characteristic	Values
Patient demographics	
Age (y), mean (SD)	47.2 (25.7)
Sex, n (%)	
Female	69,015 (51.6)
Male	64,666 (48.4)
Unknown	67 (0.0)
Race, n (%)	
Asian	22,994 (17.2)
Black	6418 (4.8)
Native American	414 (0.3)
Pacific Islander	2469 (1.9)
Unknown	1564 (1.2)
White	55,948 (41.8)
Other or multiple	43,939 (32.9)
Ethnicity, n (%)	
Hispanic or Latino	35,132 (26.3)
Non-Hispanic	96,965 (72.5)
Unknown	1649 (1.2)
Insurance, n (%)	
Commercial	48,894 (36.6)
Medicare	30,357 (22.7)
Medicaid or Medi-Cal	33,632 (25.2)
Unknown	11,617 (8.7)
Other	9242 (6.9)
Primary language, n (%)	
English	110,165 (82.4)
Spanish	16,371 (12.2)
Chinese languages	2257 (1.7)
Southeast Asian languages	1513 (1.1)
Other	3339 (2.5)
Interpreter services, n (%)	
Interpreter needed	20,483 (15.3)
No interpreter needed	113,162 (84.6)
Unknown	103 (0.1)
Encounter characteristics	
Total patient encounters, n	244,255
Encounters per resident, mean (95% CI)	3,940 (3,794-4,086)
Admission rate, %	35.7
Length of stay (h), median (IQR)	4.8 (3.1‐7.2)
Emergency Severity Index, n (%)	
1	2759 (1.2)
2	57,870 (24.1)
3	157,556 (65.7)
4	19,766 (8.2)
5	1894 (0.8)
Missing	4410 (1.8)

### Topic Exposure Progression and Interresident Variation

**Figure 1.**
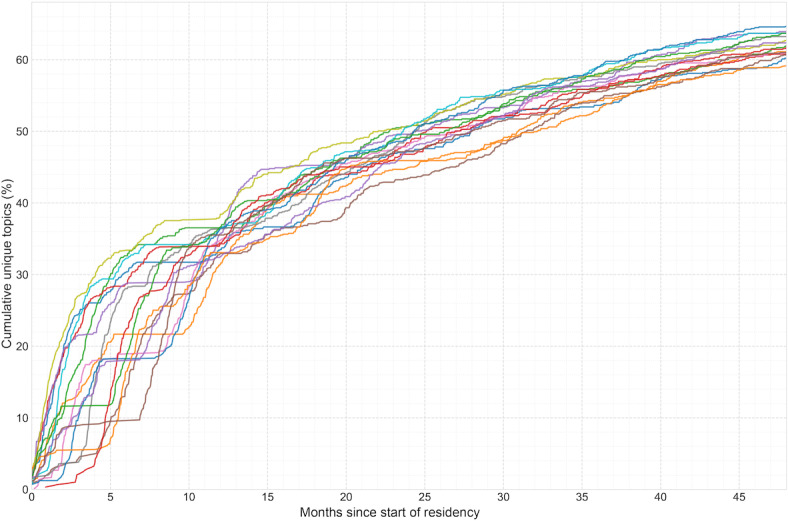
Cumulative clinical topic exposure curve for the emergency medicine graduating class of 2020 at Stanford Hospital, 2016‐2020 (n=15). The x-axis represents months of training, and the y-axis represents the mean cumulative number of unique clinical topics from the Model for Clinical Practice of Emergency Medicine encountered by residents. The curve does not reach the total of 895 Model for Clinical Practice of Emergency Medicine topics, indicating that no resident achieved 100% topic exposure during their training at the primary academic site.

EM residents demonstrated a clear, progressive acquisition of clinical topic exposure throughout training (Figures1^4^). The most rapid new topic exposure occurred during postgraduate year 1 (PGY1), with residents encountering a mean of 376.7 (42.1%) unique topics of the 895 MCPEM subcategories. However, PGY1 was also the year with the greatest interresident variability in topic coverage (coefficient of variation [CV]=9.1%). As residents progressed through training, the variation in total topic exposure decreased (PGY4 CV=2.8%).

Exposure coverage continued to increase in subsequent years, reaching a mean of 447.6 (50%) of MCPEM topics in PGY2, 515.0 (57.5%) in PGY3, and 565.9 (63.2%) in PGY4. Exposure plateaus, defined as periods with minimal new topic exposure, typically occurred in the fourth year of training; for example, the Class of 2023 reached plateaus at a mean of 39.8 (SD 3.0) months (mean 3268 encounters). However, individual variation was substantial: some residents plateaued as early as 38.2 months (2742 encounters), while others continued to encounter new topics through their final month, with final topic coverage ranging from 59.44% (532/895) to 67.26% (602/895) of all possible topics among PGY4 residents.

**Figure 2.**
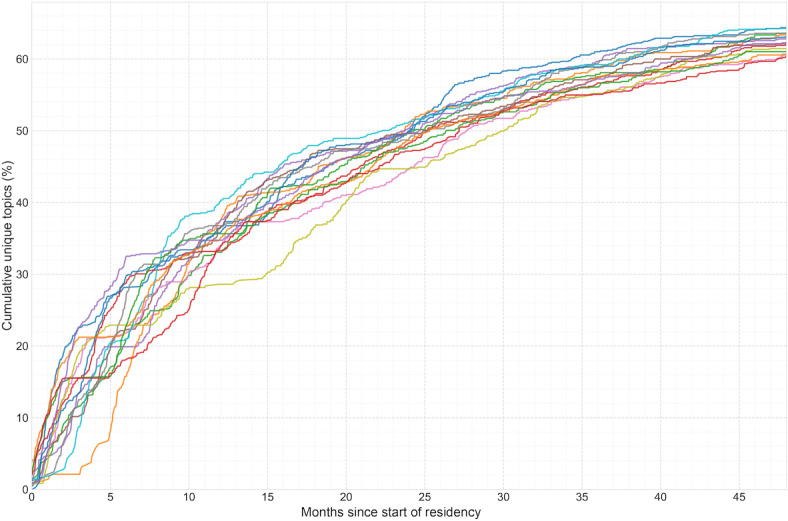
Cumulative clinical topic exposure curve for the emergency medicine graduating class of 2021 at Stanford Hospital, 2017‐2021 (n=16). The x-axis represents months of training, and the y-axis represents the mean cumulative number of unique clinical topics from the Model for Clinical Practice of Emergency Medicine encountered by residents. The curve does not reach the total of 895 Model for Clinical Practice of Emergency Medicine topics, indicating that no resident achieved 100% topic exposure during their training at the primary academic site.

**Figure 3.**
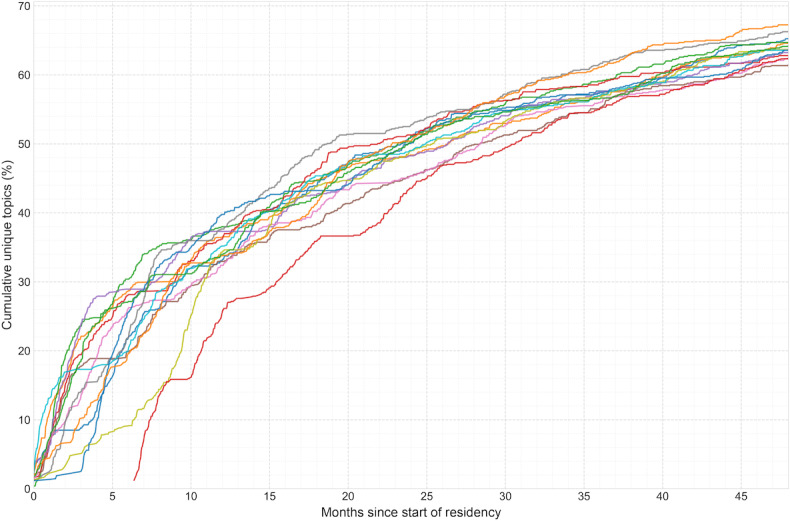
Cumulative clinical topic exposure curve for the emergency medicine graduating class of 2022 at Stanford Hospital, 2018‐2022 (n=15). The x-axis represents months of training, and the y-axis represents the mean cumulative number of unique clinical topics from the Model for Clinical Practice of Emergency Medicine encountered by residents. The curve does not reach the total of 895 Model for Clinical Practice of Emergency Medicine topics, indicating that no resident achieved 100% topic exposure during their training at the primary academic site.

**Figure 4.**
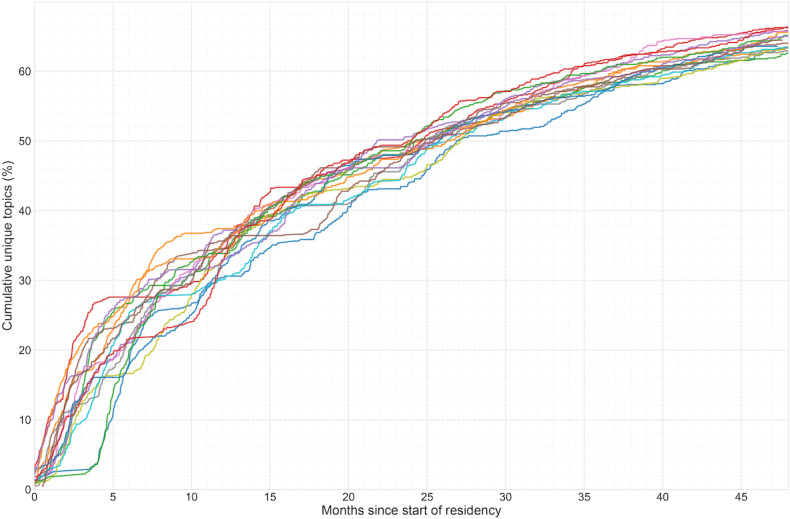
Cumulative clinical topic exposure curve for the emergency medicine graduating class of 2023 at Stanford Hospital, 2019‐2023 (n=16). The x-axis represents months of training, and the y-axis represents the mean cumulative number of unique clinical topics from the Model for Clinical Practice of Emergency Medicine encountered by residents. The curve does not reach the total of 895 Model for Clinical Practice of Emergency Medicine topics, indicating that no resident achieved 100% topic exposure during their training at the primary academic site.

### Progression of Clinical Workload and Complexity

In parallel with increasing topic exposure, residents demonstrated a progressive increase in their clinical workload and the complexity of cases managed (Table 2). Annual case volumes more than tripled from PGY1 (mean 445.7 encounters) to PGY4 (mean 1528.4 encounters). This growth in volume was accompanied by increasing case acuity, as mean ESI scores progressively decreased from 2.94 in PGY1 to 2.79 in PGY4. Correspondingly, the proportion of high-acuity patients (ESI 1‐2) managed by residents increased from 16% (4374/27,340) in PGY1 to 30.91% (9418/30,466) in PGY4, and admission rates rose from 31% (8475/27,340) to 37.50% (11425/30,466) over the same period. Notably, the CV for both clinical volume and admission rates followed a U-shaped pattern, decreasing from PGY1 to PGY3 before increasing again in PGY4.

**Table 2.** Progression of clinical experience by postgraduate year (PGY) for emergency medicine residents (N=62), Stanford Hospital, 2016 to 2023.

	PGY1	PGY2	PGY3	PGY4
Annual clinical volume and complexity				
Number of encounters, mean (95% CI)	445.7 (417.6‐473.7)	772.1 (738.5‐805.8)	1193.4 (1138.5‐1248.2)	1528.4 (1429.1‐1627.8)
ESI^a^ score, mean (95% CI)	2.94 (2.93‐2.96)	2.87 (2.85‐2.88)	2.85 (2.84‐2.87)	2.79 (2.76‐2.81)
High-acuity cases (ESI 1‐2), n/N (%)	4374/27,340 (16)	5575/27,479 (20.2)	7398/30,101 (24.5)	9418/30,466 (30.9)
Admission rate, % (95% CI)	31.0 (29.7‐32.4)	38.3 (37.3‐39.4)	35.5 (34.6‐36.3)	37.5 (36.3‐38.8)
Cumulative topic exposure				
Cumulative unique MCPEM^b^ topics covered, mean (95% CI)	376.7 (367.9‐385.5)	447.6 (442.2‐453.0)	515.0 (510.9‐519.0)	565.9 (561.9‐569.9)
Cumulative total percentage of MCPEM covered, %	41.1	50	57.5	63.2
New topics encountered each year, mean (SD)	376.7 (28.4)	71.0 (18.1)	67.3 (14.1)	50.9 (13.3)
Interresident variability (CV^c^), %				
CV for clinical volume	24.6	17	18	25.4
CV for admission rate	16.4	11.1	9.5	13.4
CV for topic coverage	9.1	4.7	3.1	2.8

a ESI: Emergency Severity Index.

b MCPEM: Model for Clinical Practice of Emergency Medicine.

c CV: coefficient of variation.

### Distribution of Clinical Experiences

Residents’ exposure to the 895 distinct clinical topics followed a consistent pattern—they encountered a small core of presentations (n=49, 5.5%) more than 100 times each, a larger set (n=284, 31.7%) between 10 and 100 times, and the majority (n=562, 62.9%) fewer than 10 times. Topic distribution showed moderate inequality (mean Gini coefficient=0.611), with consistency between the graduating classes (*P=*.61). High-acuity case exposure was more unequally distributed (Gini=0.292) than the overall case volumes (Gini=0.117).

## Discussion

### Principal Findings

Our analysis of 62 EM residents across 4 years of training revealed distinct and progressive patterns in the arc of their clinical experience. Using a novel NLP methodology on over 244,000 clinical encounters, we found that residents demonstrated a rapid acquisition of topic exposure in their first year, which continued, albeit at a slower rate, deep into their fourth year. Importantly, this continued exposure occurs in the context of increasing clinical complexity and, based on our program’s structure, escalating supervisory responsibility. These findings provide empirical evidence that can inform the national debate on the optimal length of EM training and highlight the potential for data-driven, precision education.

### Implications for Competency-Based Medical Education

The observed pattern of topic exposure, rapid initial acquisition followed by a plateau, aligns with the power-law “experience curves” documented in medical education by Pusic et al [21] However, it is critical to note that case exposures are merely the substrate for learning and are not all equal in value toward developing competence. The conversion of these experiences into durable competence is a complex process mediated by the *deliberate* components of the theory of deliberate practice by Ericsson [22], such as feedback, reflection, and coaching [22-24]. Indeed, recent research has shown that even established competency measures, such as milestones, may not directly correlate with early-career patient outcomes, cautioning against a simple equation of more exposure with more competence [25]. Therefore, the primary value of the exposure data we present lies in its ability to serve as a powerful objective input for these established educational frameworks. At our institution, for example, these components are formalized through a resident coaching program and a quarterly Clinical Competency Committee, systems for which this objective exposure data can provide a more precise foundation for assessment and goal-setting, and tracking progress toward the Accreditation Council for Graduate Medical Education milestones [2,26].

### Informing the Debate on Training Duration

A central question for the EM as a specialty is whether a 3- or 4-year training model is optimal. Our data provide empirical evidence relevant to this debate. The finding that residents continue to acquire a mean of 50.9 new core topics in their PGY4 year, representing a 9.9% (51/515) increase over PGY3, suggests that the fourth year offers more than just redundant experience. This quantitative increase is accompanied by a significant qualitative shift—PGY4 residents manage a higher proportion of high-acuity patients (ESI 1‐2) and cases requiring hospitalization. This exposure to a more complex and challenging case mix, as supported by work from Lam et al [10] and Zhou et al [27], is critical for developing the advanced diagnostic reasoning necessary for independent practice.

### Understanding Interresident Variation

Our analysis also revealed significant interresident variation, particularly in the timing of exposure plateaus and in the experience with high-acuity cases. This aligns with findings from other specialties and supports a more personalized view of competency development [28]. This variability is likely to be driven by a combination of systemic factors and resident choices. We propose framing this variability within the concept of “warranted versus unwarranted variation” as described by Holmboe and Kogan [29]. While some variation is an expected and even desirable feature of individualized learning, our NLP-based tool provides a mechanism for programs to identify potentially unwarranted gaps in exposure to core experiences. Notably, the decreasing CV in total topic coverage from PGY1 (9.1%) to PGY4 (2.8%) suggests that a longer training duration may lead to a more standardized and equitable clinical experience among graduates.

The U-shaped pattern observed in the variability of clinical volume and admission rates is an intriguing finding. We hypothesize that this reflects the evolving roles within our program—PGY1 residents have variable schedules due to numerous off-service rotations; PGY2 and PGY3 residents assume more structured supervisory roles within a pod system, which may standardize their workflow and decrease variability; finally, in PGY4, residents are granted greater autonomy to run a pod independently while also supervising junior residents, potentially allowing individual practice styles to reemerge at increasing variability.

### Bridging the Curriculum-Practice Gap and Methodological Advantages

A key finding of this study is that even after 4 years, residents were not exposed to over a third of the topics listed in the MCPEM (329/895, 36.76%). This highlights the fundamental tension between the prescribed curriculum and the reality of clinical practice. Achieving 100% topic coverage is likely an unachievable goal for any single residency program. The true aim of training is not encyclopedic exposure but rather developing the core competencies and adaptive expertise required for safe, independent practice and effective lifelong learning. Our NLP-based methodology offers a powerful, dual-pronged approach to address this gap. At the local program level, it serves as a diagnostic tool, enabling educators to identify and amend exposure gaps through targeted interventions, such as simulation. More broadly, the scalability of this approach presents an opportunity to transform the specialty’s understanding of its own work. If applied across multiple institutions, this method could create a dynamic, data-driven map of the actual clinical practice of EM, providing an evidence base for future revisions of the MCPEM. This would allow the model to better reflect the true prevalence and complexity of conditions encountered in contemporary practice, ensuring a more authentic alignment between what is taught, what is tested, and what is practiced. Nonetheless, there will likely always remain a set of high-acuity, low-frequency conditions for which training programs must prescribe exposure through didactics, as real-world encounters will be too rare to ensure universal competence.

A key advantage of this methodology is its practical implementation. Traditional NLP pipelines often require a substantial upfront investment in manual data annotation for model training and specialized expertise. In contrast, our retrieval-augmented generation approach takes advantage of a pretrained large language model that requires no model-specific training, dramatically reducing the implementation complexity. To offer a concrete sense of financial feasibility, the total cost for the application programming interface calls to process all 244,255 clinical encounters for this study was approximately US $180, demonstrating the financial accessibility of this approach for programs seeking to adopt data-driven educational strategies. Framing our pipeline in IR and IE terms also clarifies where most misclassifications arise—either from retrieval scope or from information-extraction phenomena such as negation or temporality—providing a useful structure for future error analysis and comparison to traditional rule-based clinical NLP systems.

### Limitations

This study has several limitations. As a single-institution study, our findings may not be generalizable to programs with a 3-year training format, or to institutions operating in different clinical settings. A primary limitation is that our analysis excludes data from additional training sites, which constitute a significant proportion of resident training (approximately 30%‐35% of ED time, depending on the PGY level), and from off-service rotation where key procedural and critical care exposures occur (eg, anesthesia, intensive care unit, etc). The training period for our cohorts also overlapped with the COVID-19 pandemic, which may have influenced case volumes and mix, although the remarkable consistency of exposure patterns we observed across the 4 classes mitigates this concern. Finally, although our NLP approach achieved high accuracy in classifying clinical encounters (89.7% agreement with physician review), this methodology relies on the comprehensiveness of resident documentation, which may vary between individuals and over time.

In conclusion, our analysis reveals both consistent patterns in resident clinical exposure and substantial individual variation in topic exposure trajectories. The finding that many residents continue to encounter new clinical topics into their fourth year provides empirical evidence for the potential educational value of a 4-year training model. NLP of clinical documentation offers EM programs a powerful and accessible tool to objectively measure and optimize resident clinical experiences based on actual exposure patterns, moving beyond traditional metrics to foster a more precise, data-informed, and equitable approach to graduate medical education.

## Supplementary material

10.2196/82326 Multimedia Appendix 1 Technical description of the natural language processing pipeline, validation procedures, and statistical analysis methods.

## Abbreviations

CV: coefficient of variation
ED: emergency department
EM: emergency medicine
ESI: Emergency Severity Index
IE: information extraction
IR: information retrieval
MCPEM: Model for Clinical Practice of Emergency Medicine
NLP: natural language processing
PGY: postgraduate year
