# Supplementary material for: Quantifying Emergency Medicine Residency Learning Curves Using Natural Language Processing: Retrospective Cohort Study
Source: JMIR Med Educ. 2025 Dec 9;11:e82326. doi: 10.2196/82326 (PMC12688050; doi:10.2196/82326)
Supplement: Multimedia Appendix 1 [file mededu-v11-e82326-s001.docx]

**Supplemental Digital Appendix 1**

**1. Natural Language Processing (NLP) Implementation**

**1.1 NLP Pipeline Architecture**

Our approach combines retrieval-augmented generation (RAG) with a multi-stage classification framework to analyze clinical documentation. The pipeline processes clinical notes through the following stages:

1. **Preprocessing**: Clinical notes are segmented into relevant sections (history of present illness, medical decision making, and ED course), followed by standardization of medical abbreviations and removal of formatting artifacts.
2. **Concept Identification**: Google Gemini 1.5 Flash-002 (training cutoff: September 2024) identifies key clinical features, differential diagnoses considered, and critical actions taken from the preprocessed notes.
3. **Systematized Nomenclature of Medicine – Clinical Terms (SNOMED CT) Concept Mapping**: Identified clinical concepts are mapped to SNOMED CT CORE (Clinical Observations Recordings and Encoding) Problem List Subset using a hybrid search approach combining embedding similarity and BM25 algorithms.
4. **Emergency Medicine Topic Classification**: SNOMED CT concepts are mapped to topics from the 2022 Model for Clinical Practice of Emergency Medicine, which serves as our primary unit of analysis.

The developed NLP pipeline leverages two recent advances in medical coding:

- Klang et al.’s (2024) retrieval-augmented generation approach, which outperforms human coders in ED settings
- D'Oosterlinck et al.’s (2024) Infer-Retrieve-Rank framework for extreme multi-label classification

**1.2 Model Configuration**

We utilized Google Gemini 1.5 Flash-002 accessed through Google’s Vertex AI API (Application Programming Interface) with the following inference parameters:

- Temperature: 0.1 (low temperature to ensure consistent, deterministic outputs)
- Top-p: 0.8
- Top-k: 40
- Max tokens: 3000

Access was managed through an institutional HIPAA (Health Insurance Portability and Accountability Act) compliant instance to ensure compliance with data privacy requirements.

**1.3 Concept Mapping Approach**

We implemented a two-stage approach for mapping clinical documentation to SNOMED CT concepts:

1. **Initial concept extraction**: The large language model (LLM) analyzed clinical documentation to identify key medical conditions, procedures, and diagnoses.
2. **Concept mapping**: Identified concepts were matched to SNOMED CT CORE Problem List Subset (approximately 17,000 frequently-used clinical concepts) using:
   - Embedding similarity search using all-MiniLM-L6-v2 as the embedding model
   - BM25 (Best Match 25) lexical search algorithm for text matching
   - A second LLM pass for final concept selection with additional context

We chose the SNOMED CT CORE Problem List Subset over the full SNOMED CT terminology (>360,000 concepts) to balance comprehensive clinical coverage with computational efficiency. The CORE subset covers approximately 95% of usage volume across major healthcare institutions.

**1.4 Topic Mapping Validation**

To validate our classification approach, four emergency physicians reviewed a random sample of 420 encounters and verified the topic assignments. Inter-rater reliability was substantial (prevalence-adjusted-bias-adjusted kappa = 0.71). Human raters agreed with 89.7% of the reviewed LLM classifications.

**1.5 Validation Methodology**

To validate our NLP-based topic classification approach, we conducted a comprehensive evaluation involving human expert review:

1. **Sample Selection**: A random sample of 500 encounters was selected for manual review. This sample size was determined using:
   - A confidence level of 95%
   - A margin of error of 5%
   - An estimated agreement rate of 85% based on preliminary testing
2. **Review Process**: Four emergency physicians with clinical expertise independently reviewed the sampled encounters. Each reviewer was provided with:
   - The original clinical documentation
   - The NLP-generated topic classifications
   - A rating form to indicate agreement/disagreement with each classification
3. **Inter-rater Reliability**: Agreement between human raters was assessed using prevalence-adjusted-bias-adjusted kappa (PABAK), which yielded a value of 0.71, indicating substantial reliability.
4. **Validation Results**: Human raters agreed with 89.7% of the LLM-generated classifications, confirming the high accuracy of our automated approach.

This validation process provided strong evidence for the reliability of the NLP pipeline in classifying emergency medicine resident clinical experiences according to the Model for Clinical Practice of Emergency Medicine taxonomy.

**2. Data Processing Details**

**2.1 Data Sources**

Data was extracted from Stanford’s Clinical Data Repository, which contains de-identified patient data from the electronic health record. We focused on extracting:

- Patient demographics (age, gender, race, ethnicity, primary language)
- Insurance status and interpreter needs
- Emergency Severity Index (ESI) triage level
- Chief complaint
- ED length of stay
- Disposition status
- Relevant sections of clinical documentation (history of present illness, medical decision making, ED course)

Protected health information had been previously removed through institutional de-identification processes.

**2.2 Exclusion Criteria Details**

We excluded encounters where:

- The resident was an off-service resident (not training in emergency medicine)
- Documentation was insufficient (e.g., left without being seen)
- Some highly sensitive or identifiable encounters are excluded from Stanford Research Repository (STARR)

**2.3 Topic Acquisition Definition**

For the purposes of this study, a “topic” was defined as a specific clinical concept from the 2022 Model for Clinical Practice of Emergency Medicine (MCPEM). A resident was considered to have “acquired” a topic after their first documented encounter with that topic.

Specifically:

1. Each clinical encounter was processed through the NLP pipeline to identify all topics addressed in that encounter
2. Topics were recorded as “new” if they had not been previously encountered by that resident
3. Cumulative unique topics were tracked chronologically for each resident

This first-exposure definition was chosen based on prior research showing that initial clinical exposure establishes foundational knowledge, with subsequent exposures reinforcing and building upon this base. While a single exposure may not confer mastery, it represents an important milestone in skills acquisition and development of clinical reasoning.

**2.4 Processing Optimizations**

The pipeline was implemented with several optimizations for processing large clinical datasets:

- Batch processing with configurable batch sizes (128-2048 records)
- Asynchronous processing to manage API rate limits and throughput
- Memory management with explicit garbage collection and GPU (Graphics Processing Unit) memory cleanup between batches
- Checkpoint-based processing to enable recovery from failures
- Machine-specific configuration profiles to optimize processing based on available resources

**3. Statistical Analysis Methods**

**3.1 Learning Curve Analysis**

We modeled resident learning by analyzing:

- Individual learning curves (cumulative unique topics versus time)
- Topic acquisition rates at 6-month intervals
- Learning efficiency calculated as monthly rates of new topic exposure

Plateau detection was implemented using a sliding window approach with the following parameters:

- Window size: 100 patient encounters
- Step size: 1 encounter
- Plateau threshold: <1 new topic per 100 patients
- Consecutive windows: 3 windows below threshold

For each resident, we:

1. Sorted encounters chronologically
2. Tracked cumulative unique topics and new topics for each encounter
3. Calculated topic acquisition rates in sliding windows of 100 encounters
4. Identified the first point at which three consecutive windows had acquisition rates below 1 new topic per 100 patients
5. Recorded the corresponding time (in months of training), encounter count, and cumulative topics at plateau

This approach was determined by consensus among study authors as a robust method to identify when a resident's topic acquisition had substantively slowed.

**3.2 Distribution Analysis**

Distribution of learning opportunities was assessed using:

- Gini coefficients for case acuity, topics, and volumes
- Coefficients of variation to quantify within-class consistency
- Kruskal-Wallis tests with eta-squared effect sizes to evaluate between-class differences

**3.3 Clinical Complexity Progression**

Clinical complexity progression was analyzed using mixed-effects models to account for repeated measures. We incorporated:

- ESI scores
- Admission rates
- Length of stay

as metrics of clinical complexity.

**4. Sample Prompts and Outputs**

**4.1 Initial Concept Extraction**

The NLP pipeline processes clinical documentation through multiple stages. Below is a de-identified example showing the progression from clinical note to topic classification. Note that the model output is set up to be in JSON (Javascript Object Notation) to allow standardized processing. CUIs are Concept Unique Identifiers from SNOMED-CT.

**Sample Clinical Note (Excerpt):**

HPI: 36-year-old male presenting for evaluation of acute abdominal pain for one day.

He has a complicated past medical history significant for rectal cancer, S/P surgical

resection with ileostomy and subsequent loop takedown. He has undergone cycles of

radiation therapy in the past for the colon cancer. He presents to the ED tonight

complaining of acute abdominal pain, described as moderate-severe intensity, located

in the left lower quadrant area of the abdomen, non-radiating, not associated with

nausea or vomiting, positive diarrhea today, no fevers or chills.

**Prompt:**

"""You are a medical term extractor. Extract the most important medical terms from this Emergency Department note.

Your response must be a valid JSON object with only a "medical_terms" array.

Clinical Note:

{note}

RESPONSE FORMAT:

{{

"medical_terms": [

"term1",

"term2",

"term3"

]

}}

REQUIREMENTS:

1. Response must be ONLY valid JSON

2. Each term must be under 50 characters

3. Maximum 10 most important terms

4. Focus on diagnoses, symptoms, and findings

5. Exclude administrative details

6. Keep total response under 1000 characters

DO NOT include any explanations or additional text outside the JSON."""

**Initial Gemini Response (Medical Term Extraction):**

{

"medical_terms": [

"acute abdominal pain",

"rectal cancer",

"left lower quadrant pain",

"diarrhea",

"ileostomy",

"colon cancer",

"radiation therapy",

"chemotherapy",

"abdominal pain",

"itchy rash"

]

}

**4.2 SNOMED CT Concept Mapping**

**Hybrid Search Results (Term to SNOMED CT mapping):**

[

{

"term": "left lower quadrant pain",

"concept": "Left lower quadrant pain",

"cui": "C0238551",

"score": 1.0

},

{

"term": "diarrhea",

"concept": "Diarrhea symptom",

"cui": "C0011991",

"score": 1.0

},

{

"term": "radiation therapy",

"concept": "Radiation therapy complication",

"cui": "C0393386",

"score": 1.0

},

{

"term": "chemotherapy",

"concept": "Chemotherapy",

"cui": "C3665472",

"score": 1.0

},

{

"term": "abdominal pain",

"concept": "Abdominal pain",

"cui": "C0000737",

"score": 1.0

}

]

**Gemini CUI Selection Prompt:**

"""Select the most appropriate CUIs from the list below based on this clinical note. Keep response VERY CONCISE.

Note: {note}

Available CUIs:

{results_text}

REQUIREMENTS:

1. Response must be valid JSON

2. Include ONLY selected_cuis array

3. Each CUI must be from the list above

4. Maximum 5 CUIs

5. Each CUI must have rank (1=most important)

6. Keep total response under 1000 characters

Example:

{{

"selected_cuis": [

{{"cui": "C0027051", "rank": 1}},

{{"cui": "C0008031", "rank": 2}}

]

}}

CRITICAL:

- Only use CUIs from list above

- Maximum 5 CUIs

- Include rank for each CUI

- Keep response under 1000 characters

- No explanations or extra fields

"""

**Gemini CUI Selection Response:**

{

"selected_cuis": [

{

"cui": "C0000737",

"rank": 1

},

{

"cui": "C0238551",

"rank": 2

},

{

"cui": "C0011991",

"rank": 3

}

}

]

}

**4.3 Emergency Medicine Topic Classification**

**Final Topic Classifications:**

signs_symptoms_and_presentations_pain_abdominal_pain

signs_symptoms_and_presentations_general_diarrhea

hematologic_and_oncologic_disorders_oncologic_emergencies_chemotherapy_complications

This example demonstrates how our NLP pipeline processes clinical documentation from the initial extraction of medical terms to mapping these terms to SNOMED CT concepts and finally classifying them according to the Model for Clinical Practice of Emergency Medicine topics. The pipeline successfully identified key clinical elements (abdominal pain, left lower quadrant pain, diarrhea) as well as relevant clinical context (history of cancer treatment, complications from therapy).

**5. Topic to SNOMED CT Mapping**

The mapping between SNOMED CT concepts and the Model for Clinical Practice of Emergency Medicine topics was developed using a combination of LLM suggestions and manual expert review. Below is a sample of this mapping:

| **SNOMED CT Concept** | **CUI** | **Emergency Medicine Topic** |
| --- | --- | --- |
| Abdominal pain | C0000737 | signs_symptoms_and_presentations_pain_abdominal_pain |
| Left lower quadrant pain | C0238551 | signs_symptoms_and_presentations_pain_abdominal_pain |
| Diarrhea symptom | C0011991 | signs_symptoms_and_presentations_general_diarrhea |
| Chemotherapy | C3665472 | hematologic_and_oncologic_disorders_oncologic_emergencies_chemotherapy_complications |
| Radiation therapy complication | C0393386 | hematologic_and_oncologic_disorders_oncologic_emergencies_radiation_complications |
| Chest pain | C0008031 | signs_symptoms_and_presentations_pain_chest_pain |
| Shortness of breath | C0013404 | signs_symptoms_and_presentations_respiratory_dyspnea |
| Fever | C0015967 | signs_symptoms_and_presentations_general_fever |
| Headache | C0018681 | signs_symptoms_and_presentations_pain_headache |
| Hypertension | C0020538 | cardiovascular_disorders_arterial_hypertension |
| Atrial fibrillation | C0004238 | cardiovascular_disorders_dysrhythmias_atrial_fibrillation |
| Asthma exacerbation | C0349790 | pulmonary_disorders_obstructive_lung_disease_asthma |
| Diabetic ketoacidosis | C0011880 | endocrine_disorders_glucose_metabolism_disorders_diabetic_ketoacidosis |
| Pneumonia | C0032285 | pulmonary_disorders_infectious_disorders_pneumonia |
| Acute myocardial infarction | C0027051 | cardiovascular_disorders_ischemic_heart_disease_acute_coronary_syndrome |

The complete mapping includes over 17,000 SNOMED CT concepts from the CORE Problem List Subset mapped to the 895 topics in the Model for Clinical Practice of Emergency Medicine.

**6. Performance Metrics and Processing Statistics**

**6.1 Pipeline Performance**

The NLP pipeline demonstrated robust performance in processing the large volume of clinical documentation. Key performance metrics include:

- **Processing Speed**: Approximately 75 notes per minute
- **Memory Usage**: Peak memory consumption of 12GB RAM (Random Access Memory) during embedding operations
- **Time to Process Full Dataset**: Approximately 54 hours for the complete dataset of 244,000+ encounters

**6.2 Success Rates**

- **Successfully Processed Entries** 96.3% of encounters were processed successfully
- **Failed Processing**: 3.7% of notes failed processing due to:
  - Malformed or incomplete documentation (3.22%)
  - No clinical note available (0.45%)

**6.3 Topic Distribution Statistics**

- **Total Unique Topics Identified**: 895 topics from the Model for Clinical Practice of Emergency Medicine
- **Average Topics per Note**: 4.3 topics identified per encounter
- **Most Common Topics**: Top 20 topics accounted for 38% of all topic occurrences
- **Least Common Topics**: Bottom 100 topics appeared in less than 0.1% of encounters each

**6.4 Pricing**

- Virtual Machine: 54 hours at ~$1/hr = ~$54
- API Calls:
  - Total average input/note: 18,132 characters
  - Total average output/note: 357 characters
  - Total input for ~250,000 notes: 4.5 billion characters
  - Total output for ~250,000 notes: 89 million characters
  - Model pricing: $0.0375/million characters input; $0.15/million characters output
  - API call cost: ~$180

**7. Computational Resources and Infrastructure**

**7.1 Hardware Configuration**

- **Primary Processing Server:** Google Cloud g2-standard-12 instance with:
  - 1x NVIDIA L4 GPU (24GB VRAM)
  - 12 vCPUs (Intel Cascade Lake)
  - 48GB RAM
  - 500GB SSD (Solid State Drive) storage

**7.2 Software Stack**

- **Core Technologies**:
  - Python 3.10
  - PyTorch 2.0
  - Google Vertex AI API
  - SentenceTransformers library
  - Pandas and NumPy for data processing
  - PyArrow for efficient data storage
- **Resource Management**:
  - Asynchronous processing with asyncio
  - CUDA acceleration for embedding generation
  - Memory-optimized data structures for large-scale processing

**7.3 Data Security and Privacy**

All processing was conducted in compliance with institutional policies for protecting patient health information:

- **Pre-processing De-identification**: All protected health information (PHI) was removed prior to NLP processing
- **Secure Infrastructure**: Processing conducted within HIPAA-compliant environments
- **Minimal Data Transfer**: Raw clinical text remained within secure institutional boundaries
- **Access Controls**: Strict authentication and authorization protocols for all research team members

The computational infrastructure was designed to balance processing efficiency with the security requirements for handling clinical data at scale.
